# Predicting changes in mandibular length and total anterior facial height using IGF-1, cervical stage, skeletal classification, and gender

**DOI:** 10.1186/s40510-015-0076-y

**Published:** 2015-04-27

**Authors:** Mohamed I Masoud, Hussain Y A Marghalani, Mohamed Bamashmous, Najlaa M Alamoudi, Douaa El Derwi, Ibrahim M Masoud, Veerasathpurush Allareddy, Nour F Gowharji

**Affiliations:** Department of Developmental Biology, Harvard School of Dental Medicine, 188 Longwood Avenue, Boston, MA 02115 USA; Orthodontics Department - Faculty of Dentistry, King Abdulaziz University, Abdullah Sulayman st, Jeddah, 22254 Saudi Arabia; Department of Orthodontics, University of Buffalo, 140 Squire Hall, Buffalo, NY 14214 USA; Department of Dental Public Health, King Abdulaziz University, Abdullah Sulayman st, Jeddah, 22254 Saudi Arabia; Henry M. Goldman School of Dental Medicine, Boston University, 100 East Newton Street, Boston, MA 02118 USA; Pediatric Dentistry Department, Faculty of Dentistry, King Abdulaziz University, Abdullah Sulayman st, Jeddah, 22254 Saudi Arabia; Public Health and Community Medicine Department, Faculty of Medicine, Cairo University, Al Kasr st, Kasralainy, Cairo 11562 Egypt; Dr. Ibrahim Masoud’s Dental Specialty Clinic, Jeddah, Saudi Arabia, Falasteen Street, Jeddah, 21426 Saudi Arabia; Department of Orthodontics, College of Dentistry and Dental Clinics, The University of Iowa, 801 Newton Road, Iowa City, 52242 IA USA; Department of Pediatric Dentistry, Tufts School of Dental Medicine, 1 Kneeland St, Boston, MA 02111 USA

**Keywords:** Mandible, IGF-1, Vertical dimension, Craniofacial biology, Developmental biology, Growth factors, Growth prediction

## Abstract

**Background:**

The purpose of this study was to predict the annual growth rate of the mandible and total anterior facial height using IGF-1 levels together with cervical stage, skeletal classification, and gender.

**Methods:**

Twenty-five orthodontic patients (12 females and 13 males) had their cervical stages, blood-spot IGF-1 levels, and cephalometric parameters measured at 1-year intervals. The number of years each patient was followed up varied between 1 and 5 years resulting in 43 12-month intervals collected from 77 observations. Descriptive, bivariate, and regression analyses were used to analyze this data.

**Results:**

The linear regression model for predicting the annual mandibular growth rate was significant at *p* < 0.01 with an *R*-square value of 0.52. We found that the average IGF-1 level for the interval, the change in IGF-1 level, and the presence of a skeletal class III pattern were statistically significant predictors of mandibular growth. The regression model for predicting the annual change in anterior facial height was significant at *p* < 0.01 with an *R*-square value of 0.42. We found that the change in IGF-1 level was the only statistically significant predictor of this outcome.

**Conclusions:**

The proposed method which combines IGF-1 levels with information that is readily available to clinicians can be used to predict the timing and intensity of the growth spurt. These factors together explain more of the observed individual variation in growth rate than any of the factors used in isolation.

## Background

Currently, several methods for predicting facial growth are in vogue [1-5]. The frequently used methods include skeletal maturation indicators (SMIs) developed by Fishman and cervical vertebral maturation (CVM) derived from hand-wrist radiographs [1-5]. IGF-1 is a mediator for growth hormone that has been shown to exhibit local and systemic influences in stimulating longitudinal bone growth [6-8]. In earlier studies, we had cross-sectionally related IGF-1 levels to both cervical stages and hand-wrist radiograph stages, with the highest IGF-1 levels being observed in the late pubertal stages [9-11]. Our prior research has shown that there is a significant positive correlation between longitudinal changes in IGF-1 levels and changes in mandibular length with significantly greater mandibular growth occurring when IGF-1 levels showed an ascending pattern and averaged over 250 μg/L over two consecutive observations [11]. To date, the simultaneous interaction between IGF-1, cervical stage, gender, and skeletal classification has not been longitudinally studied, and no method has been proposed to predict the timing and intensity of facial growth using IGF-1 controlling for these factors.

The purpose of the present study was to use linear regression models that use IGF-1 levels to predict the annual growth rate of anterior facial height as well as mandibular length while controlling for the effects of cervical stage, skeletal classification, and gender. We hypothesized that IGF-1 levels will be associated with increases in anterior facial height and mandibular length.

## Methods

### Institutional review board approval

The study protocol was approved by the institutional review board (IRB) of the Harvard Medical School and the ethical committee of King Abdulaziz University.

### Study sample and informed consent

This prospective longitudinal sample consisted of 25 subjects (13 males and 12 females). Inclusion criteria were patients who were to begin orthodontic treatment, in active treatment, or in post-treatment follow-up. Since the study involved radiographic exposure, non-orthodontic patients were not included for ethical reasons. Exclusion criteria included patients who had systemic diseases, growth abnormalities, bleeding disorders, or growth modification as part of their orthodontic treatment. Patients who satisfied the inclusion criteria and agreed to participate in the study were all included without further selection. The subjects were all mostly Mediterranean Caucasians, but interracial marriages are common in the population that was sampled. The objectives of the study were explained to the subjects, and consent forms were signed by those who agreed to participate. The study was conducted following informed consent and IRB approval.

### Sample size estimation and power analysis

A power analysis at the beginning of this study showed that we needed over 95 1-year intervals to be able to reject our null hypothesis if a negative result was found. Each 1-year interval required an observation at the beginning and end of the year for the interval to qualify. The original study sample, attrition rate, and the reasons for attrition are described in greater detail in our earlier studies [9-11]. In summary, our original sample comprised 87 subjects with the intent of following them up every 12 months (±2 months) until we collected 95 1-year intervals with the hope that even with attrition, our sample would be collected within 3 to 5 years. However, only 25 subjects showed up for the second observation, so the remaining 62 subjects were eliminated from our longitudinal sample. The sample continued to experience gradual attrition with only 6 subjects agreeing to attend their sixth observation which marked 5 years of follow-up and the end of the study. Any 1-year interval with missing data or non-diagnostic records at the beginning or end of the interval was eliminated from the study. At the end of the study, we had 43 qualifying 1-year intervals (17 female and 26 male intervals) from 77 observations with several observations serving as the end of one interval and the beginning of the next one. This meant that our sample would allow us to accept positive results but that negative results could not prove the absence of a relationship and could not be accepted.

### Patient records

As a part of the patients’ standard orthodontic records, lateral cephalometric radiographs were obtained and a questionnaire about age, puberty, and history of blood disorders was completed. Each patient had his or her lateral cephalometric radiograph, height, and weight measurements, as well as a blood-spot sample obtained within 1 week of each other for each observation. The blood-spot samples were collected using kits donated by ZRT Laboratory (Beaverton, OR, USA) and were stored in sealed plastic bags in a freezer (−18°C) for no more than 6 months. The samples were sent to ZRT Laboratory and assayed by radioimmunoassay. The patients’ cephalometric radiographs were traced using VistaDent OC (version 4.2.28, GAC TechnoCenter Orthodontic Software Solutions, Birmingham, AL, USA). Calibration was performed using a metal ruler that was positioned at the top right corner of the film and exposed together with the patient. The mandibular length was measured from the condylion to the gnathion, the total anterior facial height was measured from the nasion to the menton, and the mandibular plane was measured from the gonion to the gnathion. The skeletal classification was evaluated using the ANB angle [12]. Measurements between 0° and 4° were considered skeletal class I, measurements above 4° were considered skeletal class II, and measurements below 0° were considered skeletal class III. One examiner performed all the measurements. Although the subjects were all orthodontic patients, none of them had growth modification during the observation period and many of them were in pre- or post-treatment follow-up. To evaluate the possible effect of orthodontic treatment on the total anterior facial height, a paired *t*-test was conducted, and this showed that there was no significant change in the mandibular plane angle during the intervals (*p* = 0.582) with a mean change of −0.18° and a standard error of 0.32.

### Cervical vertebral staging

The cervical vertebrae were staged using the six stages described by Baccetti et al. [5]. Curvatures were initially called if they were equal to or greater than 1 mm deep. However, when that was done, several patients skipped stages within the 1-year follow-up period. The criteria for calling curvatures were then changed so that any curvature equal to or greater than 0.5 mm would be considered. Skipping stages were almost completely eliminated when that was done. The six cervical stages described by Baccetti et al. [5] were grouped into three groups based on the annual growth rates observed in the year following their appearance [9]: group A which included cervical stages 1 and 2 and was considered to be the pre-pubertal group; group B which included cervical stage 3 and was considered the pubertal group; and group C which included cervical stages 4, 5, and 6 and was considered to be the post-pubertal group. Both the laboratory that measured the IGF-I levels and the individual evaluating the radiographs were blinded to each other’s results to eliminate bias.

### Outcome and independent variables

The primary outcome variable of interest in the present study was annual growth rate of anterior facial height, and the secondary outcome variable was annual growth rate of the mandible. The independent (predictor) variables were average IGF-1 level and change in IGF-1 level.

### Statistical approach

The data was entered in SPSS (SPSS Version 22.0 for Windows, IBM Corp., Armonk, NY, USA) and was analyzed using the same software as well as SAS (SAS Version 9.3 for Windows, SAS Institute Inc., Cary, NC, USA). Two different multivariable linear regression models were used to predict the annual growth rate of total anterior facial height and that of the mandible. The effects of cervical stage, gender, skeletal classification, and initial length variables were adjusted in the multivariable regression models. The changes in outcomes for each one unit change in IGF-1 levels were predicted in the regression models. The total amount of variance explained by all the independent variables in the regression models was computed. Ordinary least squares approach was used to fit the multivariable linear regression models. All statistical tests were two-sided, and a *p* value of <0.05 was deemed to be statistically significant.

## Results

The final study sample comprised 13 male and 12 female subjects. The age of the study subjects ranged from 9.2 to 17.4 years. The cervical stages of the study sample at the initial encounter (T0) included stage 1 (16%), stage 2 (28%), stage 3 (12%), stage 4 (32%), and stage 5 (12%). Skeletal classifications included class I (20%), class II (64%), and class III (16%). A total of 43 1-year time intervals were available.

Results of the multivariable linear regression model examining annual mandibular growth are summarized in Table 1. Following adjustment for multiple factors including gender (male), initial mandibular length, skeletal pattern, and cervical group, the average IGF-1 levels and IGF-1 changes were significantly associated with increase in annual mandibular length. Each one unit increase in the average IGF-1 level was associated with 0.01203 unit increase in mandibular length (*p* = 0.02). Each one unit increase in the IGF-1 change was associated with 0.00864 unit increase in mandibular length (*p* = 0.01). About 52% of the total variance in the mandibular growth was explained by this multivariable regression model. Skeletal class III pattern was associated with 3.08 unit increase in mandibular length when compared to the skeletal class I group (*p* = 0.006). None of the other factors were significantly associated with increase in mandibular length. The following formula can be generated from this regression model:

**Table 1:** **Results of regression analysis to predict annual change in mandibular length**

**Independent variables**	**Parameter estimate**	**Error**	***p*** **value**
Intercept	−4.27893	6.06406	0.48
Average IGF-1 in μg/L	0.01203	0.00479	*0.02*
IGF-1 change in μg/L	0.00864	0.00316	*0.01*
Initial mandibular length in mm	0.02822	0.06394	0.66
Male gender (female is the reference group)	0.91686	0.75568	0.23
Skeletal class I (reference group)	Reference	-	-
Skeletal class II	−0.26483	0.79831	0.74
Skeletal class III	3.08272	1.06271	*0.006*
Cervical group A	0.16312	0.86144	0.85
Cervical group B	1.73768	1.10008	0.1235
Cervical group C (reference group)	Reference	-	-

*R*-square: 0.5215. *p* value for the model: 0.0007.

The annual mandibular growth rate in mm/year = −4.23 + (Average IGF-1 (in μg/L) × 0.012) + (Change in IGF-1 (in μg/L) × 0.009) + (Initial mandibular length in mm × 0.028) + (0 if the patient is a female, 0.917 if the patient is a male) + (0 if the patient is in skeletal class I, −0.265 if the patient is in skeletal class II, and 3.083 if the patient is in skeletal class III) + (0 if the patient is in cervical group C, 0.163 if the patient is in cervical group A, and 1.738 if the patient is in cervical group B).

Results of the multivariable linear regression model for predicting annual change in the total anterior face height are summarized in Table 2. Following adjustment for all available factors, each one unit IGF-1 change was associated with 0.01033 unit increase in total anterior face height (*p* = 0.02). None of the other factors were significantly associated with changes in total anterior face height. About 42% of the total variance in the changes in annual total face height was explained by this multivariable regression model. The following formula can be generated from the regression model:

**Table 2:** **Results of regression analysis to predict annual change in total anterior face height**

**Independent variables**	**Parameter estimate**	**Error**	***p*** **value**
Intercept	6.08452	8.35131	0.47
Average IGF-1 in μg/L	0.00929	0.0061	0.14
IGF-1 change in μg/L	0.01033	0.00437	*0.02*
Initial total anterior facial height in mm	−0.06665	0.07285	0.37
Male (female is the reference group)	1.75912	0.97007	0.08
Skeletal class I (reference group)	Reference		
Skeletal class II	0.30974	1.00557	0.76
Skeletal class III	1.89294	1.46272	0.20
Cervical group A	−0.88972	1.22983	0.47
Cervical group B	2.45562	1.42716	0.09
Cervical group C (reference group)	Reference		

*R*-square: 0.4228. *p* value for the model: 0.0095.

The annual total anterior facial height growth rate in mm/year = 6.08452 + (Average IGF-1 (in μg/L) × 0.00929) + (Change in IGF-1 (in μg/L) × 0.01033) + (Initial anterior facial height × −0.06665) + (0 if the patient is a female, 1.75912 if the patient is a male) + (0 if the patient is in skeletal class I, 0.30974 if the patient is in skeletal class II, and 1.89292 if the patient is in skeletal class III) + (0 if the patient is in cervical group C, −0.88972 if the patient is in cervical group A, and 2.45562 if the patient is in cervical group B).

The gonial angle, the *y*-axis, and the mandibular plane angle were not included in the multivariable regression models since they were not found to have a statistically significant impact on the outcomes.

## Discussion

The multivariable prediction equations derived in the present study can be used to predict the total anterior facial height and annual growth rate of the mandible. The change in IGF-1 establishes a pattern and gives information about whether the patient is pre- or post-pubertal. In an earlier study, we had demonstrated that patients with IGF-1 levels following an ascending pattern have significantly greater mandibular growth per year than patients with descending IGF levels [11]. However, this information can be misleading if used in isolation since IGF-1 values could show no change on a patient that is a very young, or very old, or a patient with an extended growth spurt. Each of those situations would have little or no change in IGF-1 level but would actually be in completely different stages of their development. The average IGF-1 level supplements that information since it allows us to distinguish the patients who are at or around their peak. Including the cervical stage, information was also found to add to the equations’ accuracy and variance in predicting facial growth, especially total anterior facial height. With the addition of gender and skeletal classification, we were able to increase the amount of variability in our models with the presence of a class III skeletal pattern being a statistically significant factor in predicting the mandibular growth rate. The variation in size was taken into consideration by adding the initial mandibular length and the initial total anterior facial height to the factors considered in each of their regression models. Despite our small sample size, we can be confident in accepting our positive results as being significant contributors to the equations since the statistics account for possible type I errors (false positive).

Franchi and Baccetti found that aside from the cervical stage, a patient’s gonial angle was the only cephalometric measurement that could predict how well a patient responded to functional appliance therapy [13]. Our results found that none of the cephalometric parameters evaluated including the gonial angle, mandibular plane, and *y*-axis significantly added to the ability of our regression models to predict the annual growth rate of the mandible or the anterior facial height. This, however, does not necessarily mean that there is no relationship between these measurements and a patient’s growth rate or pattern since our sample size was too small to rule out a type II statistical error (false negative).

Combining IGF-1 with the other factors improved the accuracy of the regression models and accounted for about half the observed variability. Facial growth is influenced by a variety of genetic and environmental factors and regulated by multiple local and system processes. Finding a group of factors that can account for that much of the variability is a significant step towards customizing our treatment plan decision to our individual patients and taking their biological differences into consideration.

The fact that our records were taken annually should be considered while interpreting the study results since the change in IGF-1 levels and average IGF-1 levels could be misleading if the peak in IGF-1 levels happened to occur during a 12-month interval. One would expect IGF-1 measurements taken 3 to 6 months apart to be sufficient to establish a pattern of change and should be able to give a more accurate assessment of the patient’s IGF-1 pattern and average level.

Jain and colleagues examined the association between IGF-1 levels and cervical maturation stages and found that amongst a cohort of 45 male subjects, there were highly significant associations between the two variables [4]. Ishaq and colleagues examined 120 subjects (60 males and 60 females) to correlate the levels of IGF-1 to cervical maturation stage and concluded that IGF-1 serum levels are reliable maturation indicators [14]. Our study results are consistent with these findings and further substantiate that IGF-1 levels could serve as a good diagnostic method for determining the optimal time for commencing orthodontic treatment.

All the study subjects in the present study received orthodontic treatment. Due to ethical considerations, it would be impossible to get institutional review board approval to perform a growth study involving radiographic exposure on an untreated population. Prior research has shown that fixed orthodontic appliances even with intermaxillary elastics have clinically insignificant effects on facial growth, and our findings described above are consistent with that [15,16].

The present study has several limitations, and the study results and conclusions should be interpreted keeping these in perspective. The present study used 2-D lateral cephalometric radiographs. The limitations of using 2-D images are well documented [17]. Any errors in landmark identification could yield biased estimates. The nature of the study design precludes us from clearly establishing a cause and effect relationship between the predictor and outcome variables. Only a well-designed randomized controlled trial will enable us to establish this relationship [18]. Only one biological factor was examined which accounts for the moderate correlation coefficient. However, it is still impressive that a single biological factor could account for the amount of variability explained by the multivariable regression models. Despite the fact that none of these patients had growth modification during the follow-up period, many of them were undergoing orthodontic treatment with full fixed appliances which could arguably have had an influence on their growth. As mentioned earlier, the study sample is not representative, and hence, the generalizability and external validity of the findings are questionable. The present study could have benefitted by an increased sample size and examining several more variables that could confound growth. Owing to the practical limitations on recruiting more patients to participate, we were unable to increase the sample size. This is a major limitation of the study. Finally, the prediction equations derived in the present study accounted for 52% of variance in mandibular growth and 42% of variance in anterior facial height. It is clear that a large amount of variance is explained by the predictors. Nevertheless, there could be other variables that are not captured in the models that can play a significant role in determining growth. These should be kept in perspective while treatment planning orthodontic cases.

## Conclusions

1. Linear regression models derived from the present study could help in the prediction of the timing and intensity of patients’ facial growth spurt using IGF-1 levels while controlling for cervical stage, gender, and skeletal classification.
2. Average IGF-1 for an observation period, change in IGF-1 level, and the presence of a skeletal class III pattern were statistically significant factors in the regression model for predicting changes in mandibular length.
3. Change in IGF-1 level was the only statistically significant factor in the regression model for predicting changes in total anterior facial height.
